# Pancreatic Duct Patterns in Acute Pancreatitis: A MRI Study

**DOI:** 10.1371/journal.pone.0072792

**Published:** 2013-08-28

**Authors:** Rong Peng, Xiao Ming Zhang, Yi Fan Ji, Tian Wu Chen, Lin Yang, Xiao Hua Huang, Xiao Xiao Chi

**Affiliations:** Sichuan Key Laboratory of Medical Imaging, Department of Radiology, Affiliated Hospital of North Sichuan Medical College, Nanchong, Sichuan, the People’s Republic of China; University of Szeged, Hungary

## Abstract

**Objectives:**

To study the MRI findings of the pancreatic duct in patients with acute pancreatitis.

**Materials and Methods:**

A total of 239 patients with acute pancreatitis and 125 controls were analyzed in this study. The severity of acute pancreatitis was graded using the MR severity index (MRSI) and the Acute Physiology And Chronic Healthy Evaluation II(APACHE II) scoring systems. The number of main pancreatic duct (MPD) segments visualized, and both MPD diameter and pancreatic duct disruption were noted and compared with the severity of acute pancreatitis.

**Results:**

The frequency of MPD segment visualization in the control group was higher than that in the acute pancreatitis group (p<0.05). The number of MPD segments visualized was negatively correlated with the MRSI score (p<0.05) and the APACHE II score (p<0.05). There was no difference in the MPD diameter between the acute pancreatitis and control groups or among the patients with different severities of acute pancreatitis (p>0.05). The prevalence of pancreatic duct disruption was 7.9% in the acute pancreatitis group. The prevalences of pancreatic duct disruption were 4.8% and 15.3% in the mild and severe acute pancreatitis groups based on the APACHE II score, respectively, and were 0%, 5.7% and 43.5% in the mild, moderate and severe acute pancreatitis groups according the MRSI score, respectively. The prevalence of pancreatic duct disruption was correlated with the severity of acute pancreatitis based on the APACHE II score (p<0.05) and MRSI score (p<0.05).

**Conclusion:**

The pancreatic duct in acute pancreatitis patients was of normal diameter. The number of MPD segments visualized and visible pancreatic duct disruption on MRI may be supplementary indicators for determining the severity of acute pancreatitis.

## Introduction

Acute pancreatitis is defined as the acute inﬂammation of the pancreatic gland and the surrounding tissues and is thought to result from the premature activation of pancreatic digestive enzymes [1]. In 80% of patients, acute pancreatitis is mild and resolves without serious morbidity; however, in up to 20% of patients, it is complicated by substantial morbidity and mortality [2]. Although the majority of patients have mild, self-limiting disease, some develop severe disease involving organ failure. These patients are at risk of developing complications, such as pancreatic necrosis, ﬂuid collections, pseudocysts, and pancreatic duct disruption, due to the persistent pancreatic inﬂammation [3]. Pancreatic ductal changes predict spontaneous resolution, the success of nonoperative measures, and the need for direct therapies to treat pseudocysts. Ductal changes can also identify patients with necrotizing pancreatitis who are most likely to have immediate and delayed complications [4].

Magnetic resonance imaging (MRI) is a valuable tool for the assessment of the full spectrum of pancreatic diseases and can be used to visualize and stage acute pancreatitis and its local complications [5]. The MR severity index (MRSI) has a similar value for the assessment of the severity of AP as the CT severity index (CTSI) [6]. Nonenhanced MRI is comparable to contrast-enhanced CT to assess the severity of acute pancreatitis [7]. Magnetic resonance cholangiopancreatography (MRCP) is a non-invasive imaging technique that accurately depicts the morphological features of the biliary and pancreatic ducts and is a promising alternative to diagnostic endoscopic retrograde cholangiopancreatography (ERCP) [8].

Larena et al. [8] indicated that in acute pancreatitis patients, the pancreatic duct is occasionally not visualized on MRCP because of the compression of the duct by adjacent inﬂammation and edema. Leyendecker et al. [9] and Pascual et al. [10] observed that the pancreatic duct in patients with acute pancreatitis is of normal diameter on MRCP. However, Larena et al. [8] stated that in severe cases of pancreatitis, MRCP may provide information on the status of the pancreatic duct, including information on the presence of ductal distension, disruption or leakage or the presence of an intraductal lesion that might predispose the patient to pseudocyst formation. Pancreatic duct disruption is a significant clinical event and portends a more severe clinical course after acute pancreatitis or other pancreatic injury. The identification of such a disruption can prompt the use of more aggressive medical therapy early in the disease course and might also identify those patients likely to benefit from early endoscopic intervention [11]. The prevalence of pancreatic duct disruption in patients with severe acute pancreatitis (SAP) was reported to range from 10% to 31% by EPCP or secretin MRCP [12], [13]. A retrospective study [14] reported that endoscopic treatment temporarily improved or resolved pancreatic duct disruption in 10% of patients but had a failure rate of 23%. However, to the best of our knowledge, there is no report on the relationship between the ability to visualize main pancreatic duct (MPD) segments and the severity of acute pancreatitis. The diameter of the MPD in acute pancreatitis patients is also still disputed. The prevalence of pancreatic duct disruption in all cases of acute pancreatitis detected by routine MR pancreatography is unknown.

We conducted this study to retrospectively assess the MPD in patients with acute pancreatitis and a control group using MRI. We analyzed the visualization of MPD segments, measured the diameter of the MPD, and assessed the presence or absence of pancreatic duct disruption. We then analyzed these factors in the control group and the acute pancreatitis group. We analyzed the relationship between these factors and the severity of acute pancreatitis based on the MRSI and the Acute Physiology And Chronic Healthy Evaluation II (APACHE II) scoring systems.

## Materials and Methods

### 1. Ethics Statement

This study was approved by the Institutional Review Board of the Affiliated Hospital of North Sichuan Medical College. The Ethics Committee concluded that the experimental design and the program of the study would not cause harm or risk to the subjects. Due to the retrospective nature of this study and the actual medical conditions in Nanchong, it would have been difficult to obtain informed consent from all patients involved in the study. Therefore, the Ethics Committee of our hospital waived the need for written informed consent from the participants. The Institutional Review Board of the Affiliated Hospital of North Sichuan Medical College approved obtaining the MR scans for 301 patients with acute pancreatitis and 125 control patients. The study complies with the ethical principles of the Helsinki Declaration of 1964 as revised by the World Medical Organization in Edinburgh in 2000.

### 2. Patient Population

Patients with acute pancreatitis admitted to our institution between January 2010 and April 2012 were considered for inclusion in this study. The diagnosis of acute pancreatitis was based on the presence of typical abdominal pain combined with three-fold elevation of the serum levels of amylase and lipase. The inclusion criteria for this study were as follows: (1) in-patient, (2) acute history, (3) pancreatitis at first onset, (4) three-fold elevation of the amylase or lipase level and exclusion of other causes of elevated enzyme levels, and (5) no more than a two-day interval between the abdominal MRI examination and the onset of pancreatitis. The exclusion criteria for this study were as follows: (1) hypoproteinemia; (2) intra- or retroperitoneal tumors, inﬂammation (except, of course, acute pancreatitis) or hemorrhagic diseases; (3) chronic pancreatitis; and (4) recent surgery on the biliary tract.

A total of 301 patients with acute pancreatitis met the inclusion criteria, and 62 of these patients met the exclusion criteria (4 met Condition 1; 5 met Condition 2; 5 met Condition 3; 48 met Condition 4) and were not included in the study. The final study group consisted of 239 consecutive patients. There were 128 women and 111 men with an average age of 52±15 years (range: 16–85 years). All patients underwent a clinical assessment and laboratory workup on admission.

Patients without pancreatic disorders detected by MRI who were seen during the same period served as controls. They underwent upper abdominal examinations in our hospital as part of physical examinations or assessments for other diseases. The exclusion criteria for this study were as follows: (1) pancreatic diseases; (2) cirrhosis of the liver; (3) cholecystitis; (4) biliary tract stones; (5) peritoneal effusion; and (6) abdominal or retroperitoneal tumor. In total, 125 persons made up the control group. There were 67 women and 58 men with an average age of 53±13 years (range: 12–80 years). In the control group, 51 patients had no abnormalities in the abdomen, 18 patients had hepatic cysts, 10 patients had hepatic hemangiomas and 46 patients had renal cysts on MRI.

### 3. MR Imaging Technique

All examinations were performed with a 1.5-T MR scanner with 38 mT/M gradients and a 120 mT/M-per-second slope (Signa Excite; GE Medical Systems, Milwaukee, WI, USA) using a phased-array torso-pelvis coil. The imaging sequences included two-dimensional coronal and axial single-shot fast spin-echo (SSFSE) T2-weighted imaging, axial respiratory gating fast-recovery fast-spin echo (FRFSE) T2-weighted imaging with fat suppression, fast-spoiled gradient-echo (FSPGR) T1-weighted imaging with fat suppression, axial spoiled dual gradient-echo (GRE) T1-weighted in- and out-of-phase MR imaging, axial slab three-dimensional (3D) spoiled gradient-echo (SPGR) dynamic contrast-enhanced MR imaging with fat suppression, and SSFSE radial series slab MR cholangiopancreatography (MRCP).

Coronal and axial SSFSE T2-weighted images were obtained during breath-holding with the following parameters: echo time (TE) = 90–100 ms, 2 s between slice acquisitions, section thickness = 5 mm, intersection gap = 0.5 mm, matrix = 384×224, one-half signal acquired, and field of view (FOV) = 33 cm×33 cm.

FRFSE T2-weighted images were obtained with the following parameters: repetition time (TR) ms/TE ms = 10,000–12,000/90–100, with TR determined by the frequency of respiration; section thickness = 5 mm; intersection gap = 0.5 mm; matrix = 256×192; number of signals acquired (NSA) = 3; and FOV = 34 cm×34 cm. It took approximately 3–4 min to complete the acquisition.

Radial oblique slab SSFSE images were obtained for MRCP with the following parameters: TE = 1300 ms; 6 s between image acquisitions; section thickness = 40 mm; matrix = 384×224; one-half signal acquired; and FOV = 30 cm×30 cm.

All the other routine sequences mentioned above were not used in the analysis presented in this article, and thus, we have not listed the parameters for those sequences.

It took approximately 30 min to complete all the non-contrast MRI sequences and 35 min to complete the contrast-enhanced MR imaging.

### 4. MR Image Analysis

The original MRI data were loaded onto a workstation (GE, AW 4.1, Sun Microsystems, Palo Alto, CA, USA) for review. Two observers (with 4 and 6 years of experience interpreting abdominal MR images, respectively) who were blinded to the laboratory data and clinical outcomes reviewed the MR images.

On the MR images, acute pancreatitis was defined as edematous and necrotic pancreatitis. Pancreatic necrosis was defined as a well-marginated area of signal intensity different from the signal intensity of a normal pancreas in non-enhanced image along with the absence of enhancement on enhanced imaging [14]. The severity of acute pancreatitis on MRI was evaluated with the MR severity index (MRSI), which is derived from the computed tomographic severity index [15]. Acute pancreatitis was graded as mild (0–3 points), moderate (4–6 points), or severe (7–10 points) according to the MRSI [15].

The assessment of the pancreatic duct was performed using coronal and axial SSFSE T2-weighted imaging, FRFSE T2-weighted imaging and MRCP.

Three segments of the pancreatic duct were evaluated: the head, body, and tail. The MPD was assessed using a 3-grade scale: good (whole length of the duct distinctly identifiable), fair (the duct partially obscured but identifiable), or poor (duct unidentifiable) [16]. The diameters of the pancreatic ducts were measured in the head, body, and tail at the point in each segment with the maximum diameter on axial T2WI. When it was difficult to visualize the MPD using axial T2WI, we measured it in the same manner using coronal T2WI and MRCP. Then, the maximum was used. When the pancreatic duct was not visualized in any of those sequences, the diameter of the duct was defined as 0 mm. The MPD was considered to be dilated if the basal diameter was >3 mm in patients <60 years old [17] and >3.5 mm in patients over 60 years [10]. To confidently diagnose pancreatic duct disruption, it is necessary to demonstrate all of the following features: (a) the diameter of the pancreatic necrosis area is at least 2 cm, (b) viable pancreatic tissue is present upstream (ie, toward the pancreatic tail) from the site of necrosis, (c) the MPD enters the area of fluid collection at an angle of approximately 90° [18].

### 5. The APACHE II Score

One physician with 20 years of experience with digestive diseases reviewed the medical records to determine the APACHE II score.

To calculate the APACHE II score, 12 common physiological and laboratory values–the axillary temperature, mean arterial pressure, heart rate, respiratory rate, oxygenation, arterial pH, serum sodium, serum potassium, serum creatinine, hematocrit, white blood count and Glasgow coma scale–are each assigned 0 to 4 points, with 0 points being normal and 4 points being the most abnormal. The sum of these values is added to a point weighting for patient age and a point weighting for chronic health problems (severe organ insufficiency or immunocompromised state) to arrive at the APACHE II score [19]. For all of the 239 patients, the APACHE II score was calculated from data obtained within 24 h by the physicians and nurses on duty who had no knowledge of the MRI findings. An APACHE II score of 8 was the cut-off point to differentiate between mild AP (0–7 points) and severe AP (≥8 points) [20]. De et al. [21] found that in patients with acute pancreatitis and an APACHE II score greater than eight, indicators of organ failure correlate with the development of systemic complications and with mortality.

### 6. Statistical Analysis

Data derived from the MR images were expressed as the average of the two observers’ findings. Any discrepancies in the discrete data were discussed by the two observers until a consensus was reached.

The inter-rater agreement for pancreatic duct disruption and the prevalence of MPD visualization was assessed using the kappa (k) statistic. This statistic is generally interpreted as follows: a kappa value equal to or greater than 0.81 indicates very good agreement, a kappa value ranging from 0.80 to 0.61 indicates good agreement, a kappa value ranging from 0.60 to 0.41 indicates moderate agreement, and a kappa value of less than 0.41 indicates poor agreement.

The diameter of the MPD was given as the mean±standard deviation. The Z test was used to determine the differences in the diameter of the MPD between the control and acute pancreatitis groups and between the mild acute pancreatitis and severe acute pancreatitis groups according to the APACHE II score. Analysis of variance (ANOVA) was used to assess the differences in the diameter of the MPD between the mild, moderate, and severe AP groups according to the MRSI score.

Chi-squared tests were used to assess the differences in the prevalence of the MPD visualization between the control and acute pancreatitis groups. To correlate the visualized segments of the MPD with the severity determined by the MRSI and APACHE II scores, the Z test, ANOVA and Spearman’s rank correlation coefficients were used. To correlate the prevalence of pancreatic duct disruption with the severity determined by the MRSI and APACHE II scores, the Chi-squared test and Spearman’s rank correlation coefficients were used.

The data analysis was performed using Statistical Package for Social Sciences (SPSS) for Windows (Version 13.0, Chicago, IL, USA). P values <0.05 were considered significant.

## Results

Of the 239 patients with acute pancreatitis, 111 were men and 128 were women. Their mean age was 52±15 years (range: 16–85 years). The etiology of acute pancreatitis was biliary in 48.5% (116/239), alcoholic in 7.5% (18/239), hyperlipidemic in 2.5% (6/239), and traumatic in 0.4% (1/239). Forty-one percent (98/239) of the patients did not have a specified etiology.

Based on the MRSI, 59/239 (25%), 157/239 (66%), and 23/239 (9%) of the patients had mild, moderate and severe acute pancreatitis, respectively. A total of 167 patients were diagnosed with mild acute pancreatitis (70%), and 72 patients were diagnosed with severe acute pancreatitis (30%) according to the APACHE II scoring system.

The control group included 125 outpatients (67 women and 58 men with an average age of 53±13 years, range 12–80 years).

The agreement between the two raters was generally good (Table 1) for the number of MPD segments visualized and the presence of pancreatic duct disruption on MRI.

**Table 1:** Agreement between the two raters with respect to the prevalence of the number of MPD segments visualized and the pancreatic duct disruption on MRI.

	Rater 1	Rater 2	κ values
Number of MPD segments visualized in the control group	343	352	0.67
Number of MPD segments visualized in the acute pancreatitis group	574	592	0.73
Pancreatic duct disruption in the control group	0	0	1
Pancreatic duct disruption in the acute pancreatitis group	15	19	0.68

MPD, main pancreatic duct. MRI, magnetic resonance imaging.

The depiction capability for the MPD for the 125 controls was evaluated as follows: good images were obtained for 116 of 125 for the pancreas head, 114 of 125 for the body, and 110 of 125 for the tail. Fair images were obtained for four, three, and three controls for the head, body, and tail, respectively. Poor images were obtained for five, eight, and twelve controls for the head, body, and tail, respectively. Thus, the depiction capability for the 125 controls was evaluated as fair to good for the images of the pancreas head for 120 controls (96.0%), images of the body for 117 (93.6%), and images of the tail for 113 (90.4%). The MPD depiction capabilities in patients with acute pancreatitis were evaluated as follows: good images were obtained for 190 patients of 239 for the pancreas head, for 181 patients for the body, and for 176 patients for the tail. Similarly, fair images obtained were obtained for 15, 21, and 23 patients for the head, body, and tail, respectively. Poor images were obtained for 34, 37, and 40 patients for the head, body, and tail, respectively (Table 2).

**Table 2:** Number of main pancreatic duct (MPD) segments visualized in the two groups.

	Number of MPD segments visualized			Total visualized segments
	Head	body	tail	
Control group (n = 125)	120(96.0%)	117(93.6%)	113(90.4%)	350(93.3%)
Acute pancreatitis (n = 239)	205(85.8%)	202(84.5%)	199(83.2%)	606(84.5%)

**Figure 1:**
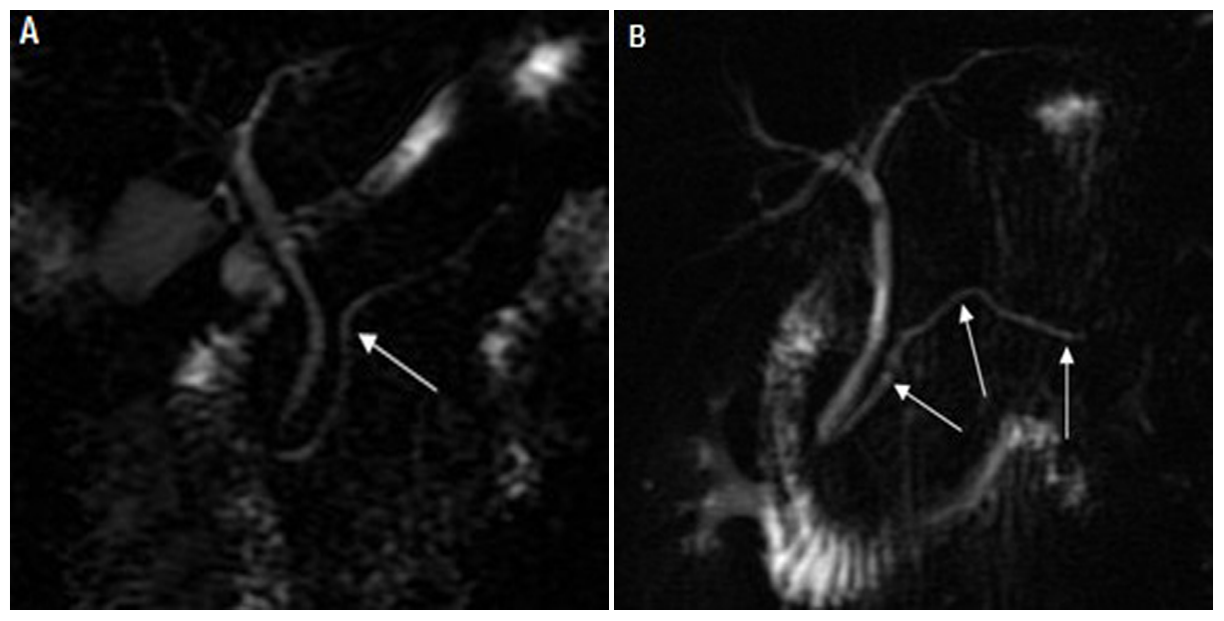
Magnetic resonance cholangiopancreatography. (A) A 42-year-old male with a normal pancreas. The visualization of the main pancreatic duct was good (arrow). (B) A 34-year-old male with edematous acute pancreatitis. The Acute Physiology And Chronic Healthy Evaluation II score was 4, and the magnetic resonance severity index was 2. The main pancreatic duct was of normal diameter. The visualization of the three segments of the main pancreatic duct was good (arrows).

**Figure 2:**
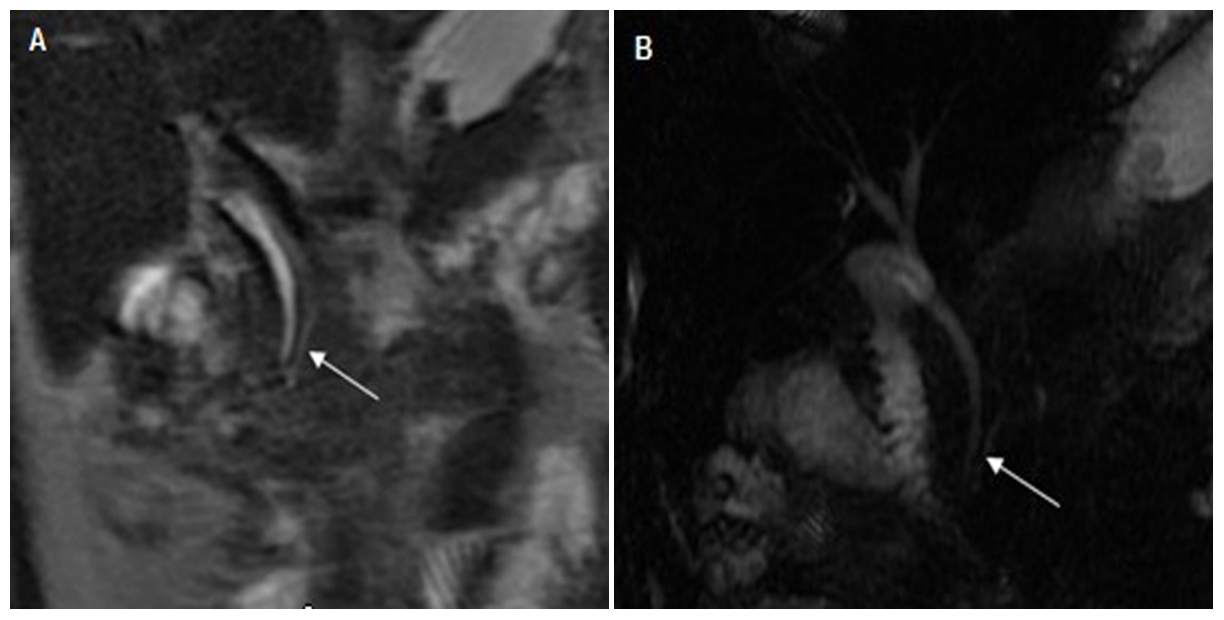
A 47-year-old female with acute pancreatitis. The Acute Physiology And Chronic Healthy Evaluation II score was 13, and magnetic resonance severity index was 10. (A) Coronal single-shot fast spin-echo T2WI and (B) magnetic resonance cholangiopancreatography show only the head of the main pancreatic duct (MPD) and the MPD visualized flair (arrows).

In the control group, 93.3% of the MPD segments (350 in total) were visualized, which was higher than the percentage for acute pancreatitis patients (84.5%, 606 segments) (p<0.001). The number of MPD segments visualized by MRI was negatively correlated with the MRSI score (r = −0.416, p<0.001) and the APACHE II score (r = −0.269, p<0.001) (Figure 1–2).

The mean diameters (±SD) of the MPDs in the two groups are shown in Table 3. Only eight patients in the acute pancreatitis group had a dilated MPD, among whom 6 patients had long-term abdominal pain and a clear diagnosis of cholecystitis or biliary tract stones. The other 2 patients had suspected pancreatic cystic tumors. However, they had not previously been diagnosed with acute pancreatitis. We therefore suspect that these eight patients may have had recurrent acute pancreatitis, chronic pancreatitis or pancreatic tumors. None of the patients in the control group had a dilated MPD. There were no differences in the diameter of the MPD between the acute pancreatitis patients and the control group (p>0.05). There were also no differences in the diameter of the MPD among the groups with different severities of acute pancreatitis according to the MRSI and APACHE II scoring systems (p>0.05) (Table 4 and Table 5).

**Table 3:** The diameters of the main pancreatic duct (MPD) in the two groups.

	MPD diameter (mm)		
	Head (Mean±SD)	Body (Mean±SD)	Tail (Mean±SD)
Control group	2.51±0.31	2.27±0.28	1.98±0.13
Acute pancreatitis group	2.52±0.32	2.24±0.31	2.02±0.17
P value	0.82	0.10	0.88

**Table 4:** The diameters of the MPD in the mild and severe acute pancreatitis groups according to the APACHE II score.

Diameter of the MPD (mm)	APACHE II score		
	Mild (Mean±SD)	Severe (Mean±SD)	P value
Head	2.50±0.33	2.55±0.27	0.19
Body	2.22±0.29	2.30±0.35	0.71
Tail	2.01±0.17	1.99±0.32	0.66

MPD, main pancreatic duct. APACHE II, the Acute Physiology And Chronic Healthy Evaluation II.

**Table 5:** The diameters of the MPD in the mild, moderate and severe acute pancreatitis groups according to the MRSI.

Diameter of the MPD (mm)	MRSI			
	Mild (Mean±SD)	Moderate (Mean±SD)	Severe (Mean±SD)	P value
head	2.51±0.36	2.52±0.30	2.42±0.27	0.53
body	2.22±0.30	2.25±0.33	2.23±0.27	0.88
tail	1.99±0.16	2.03±0.18	1.97±0.12	0.16

MPD, main pancreatic duct. MRSI, magnetic resonance severity index.

**Figure 3:**
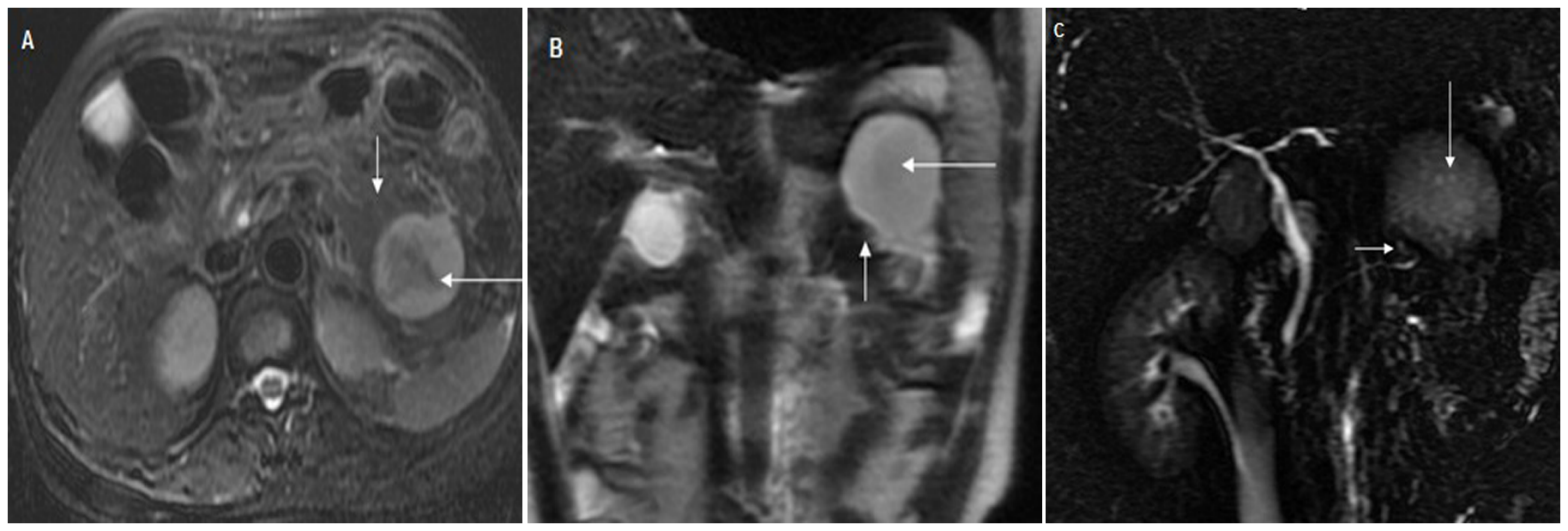
Pancreatic duct disruption in a 46-year-old female with acute pancreatitis. The Acute Physiology And Chronic Healthy Evaluation II score was 4, and the magnetic resonance severity index was 8. (A) Axial fast-recovery fast spin-echo T2-weighted image with fat suppression, (B) coronal single-shot fast spin-echo T2-weighted image and (C) magnetic resonance cholangiopancreatography show a 5-cm-diameter ﬂuid collection replacing the tail of the pancreas (long arrow). The main pancreatic duct (short arrow) enters the fluid collection.

**Figure 4:**
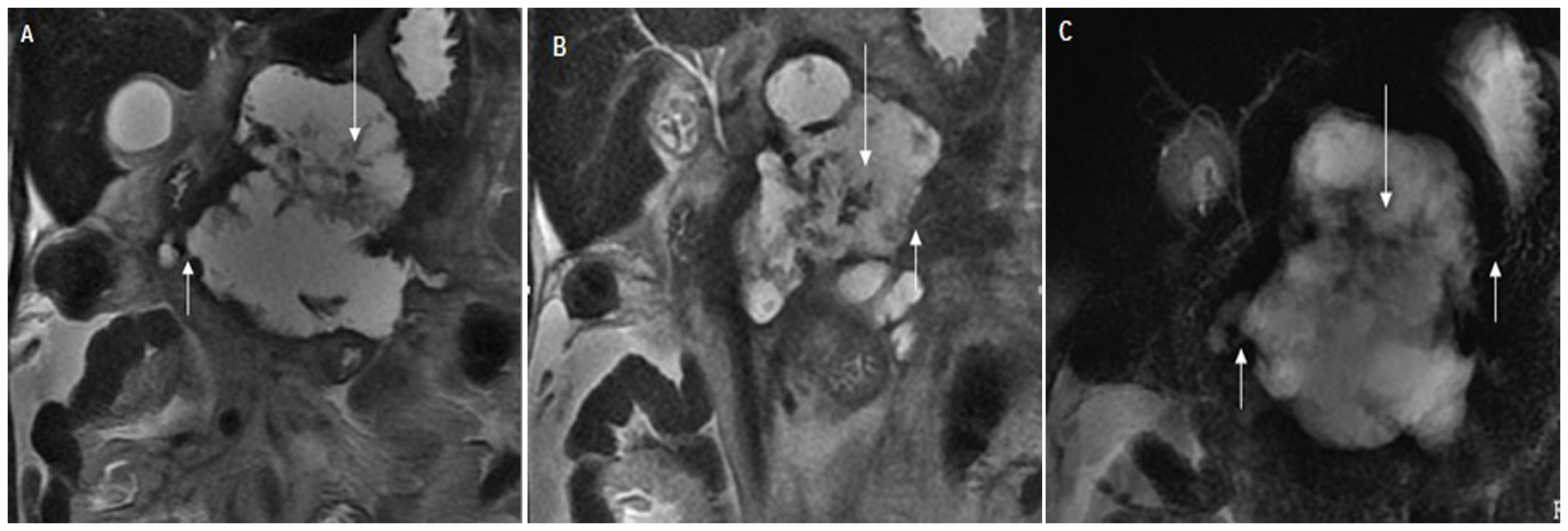
Pancreatic duct disruption in a 44-year-old female with acute pancreatitis. The Acute Physiology And Chronic Healthy Evaluation II score was 15, and the magnetic resonance severity index was 10. (A), (B) Coronal single-shot fast spin-echo T2-weighted images and (C) magnetic resonance cholangiopancreatography show a complex 8.5-cm-diameter ﬂuid collection (long arrow) in the head, body and proximal tail of the pancreas. The main pancreatic duct (short arrow) enters the fluid collection at an angle of approximately 90°.

Pancreatic duct disruption was observed in 19 patients (7.9%) (Figure 3 and Figure 4). The prevalences of pancreatic duct disruption were 0%, 5.7% and 43.5% for the mild, moderate and severe acute pancreatitis groups according to the MRSI (p<0.05 between the severe group and the non-severe group) (Table 6). The prevalences of pancreatic duct disruption were 4.8% and 15.3% in the mild and severe acute pancreatitis groups according to the APACHE II score (p<0.05) (Table 7). The frequency of pancreatic duct disruption in patients with acute pancreatitis increased with increasing APACHE II and MRSI scores. The prevalence of pancreatic duct disruption was correlated with the acute pancreatitis severity determined by the MRSI (non-severe vs. severe group, Spearman’s rank correlation coefficient, r = 0.429, p<0.001) and the APACHE II score (Spearman’s rank correlation coefficient, r = 0.178, p = 0.003).

**Table 6:** Pancreatic duct disruption and the MRSI.

Pancreatic duct disruption	MRSI score			
	Mild (n = 59)	Moderate (n = 157)	Severe (n = 23)	P value
Positive [n (%)]	0(0%)	9(5.7%)	10(43.5%)	<0.05
Negative [n (%)]	59(100%)	148(94.3%)	13(56.5%)	<0.05

MRSI, magnetic resonance severity index.

**Table 7:** Pancreatic duct disruption and the APACHE II score.

Pancreatic duct disruption	APACHE II score		
	Mild (n = 167)	Severe (n = 72)	P value
Positive [n (%)]	8(4.8%)	11(15.3%)	<0.05
Negative [n (%)]	159(95.2%)	61(84.7%)	<0.05

APACHE II, the Acute Physiology And Chronic Healthy Evaluation II.

During the hospital stay, all patients had good recoveries and were discharged after active treatment. No patient died.

## Discussion

In this study, we found that the pancreatic duct in patients with acute pancreatitis was of normal diameter. The number of MPD segments visualized in the control group was higher than that in acute pancreatitis patients. The number of MPD segments visualized by MRI was negatively correlated with the MRSI and APACHE II scores. Of the patients with acute pancreatitis, 7.9% had pancreatic duct disruption detected by MRI. Pancreatic duct disruption detected by MRI was correlated with the MRSI and APACHE II scores. The number of MPD segments visualized and the prevalence of pancreatic duct disruption on MRI in acute pancreatitis may be supplementary indicators of the severity of AP.

The rapid technological advances in MRI have led to a resurgence in the use of noninvasive imaging of the pancreaticobiliary system. MRI and MRCP are noninvasive methods that can reveal and stage acute pancreatitis and associated local complications, evaluate the etiology of this condition, allow direct visualization of the pancreatic ducts, and permit the detection of pancreatic duct disruption [1], [5], [6], [10], [11]. Endoscopic retrograde cholangiopancreatography (ERCP) remains the gold standard for the detection of pancreatic duct disruption. Currently, the role of endoscopy in the management of pancreatic duct disruption is therapeutic rather than diagnostic because ERCP is limited by an inability to access the pancreatic duct in some cases due to severe edema or a cutoff of the MPD. It appears that one of the best current tools to diagnose pancreatic duct disruption is MRCP [22]. To the best of our knowledge, the changes in the ductal system in acute pancreatitis patients are often not overt but are more subtle. Imaging techniques play an important role in differentiating these subtle changes from the normal state [23]. To identify the MPD accurately, we assessed the MPD using coronal and axial SSFSE T2-weighted imaging, FRFSE T2-weighted imaging and MRCP.

In the control group, the MPD depiction capabilities of MRCP were 96% for the head, 93.6% for the body, and 90.4% for the tail. In AP patients, the head was visualized in 85.8%, the body in 84.5%, and the tail in 83.2%. The percentages of MPD segments visualized were similar to those reported by other authors [10], [16]. We found that the percentage of MPD segments visualized in the control group (93.3%) was higher than that for acute pancreatitis patients (84.5%, p<0.05). This result may be attributed to the compression of the duct by adjacent inﬂammation and edema. Extrapancreatic fluid may also restrict the visualization of the pancreatic duct in acute pancreatitis patients [8].

The diameters found by MRI are most likely close to reality [24]. The diameter of the main pancreatic duct found in this study appears to be the same as that usually reported in the literature [24]. In the present study, the MPD was of normal diameter and was similar to that reported in the literature [8], [9], [10].

Pancreatic duct disruption is a significant clinical event that may follow severe acute pancreatitis, chronic pancreatitis, operative injury, or abdominal trauma. The frequency of MPD disruption is similar between infected and sterile necrosis [13]. Pancreatic pseudocysts and pancreatic ascites are two of the outcomes of pancreatic duct disruption [11]. Recovery after necrotizing acute pancreatitis may be incomplete, with persistent impairment of the exocrine function and ductal morphology [25]. Diagnosing pancreatic duct disruption early is very important. The prevalence of pancreatic duct disruption in severe acute pancreatitis (SAP) has been reported to range from 10% to 31% by EPCP or secretin MRCP [12], [13]. A retrospective study by Viremouneix et al. [14] reported that endoscopic treatment temporarily improved or resolved pancreatic duct disruption in 10% of patients but had a failure rate of 23%. We found that the prevalence of pancreatic duct disruption was 7.9% by routine MRI, which was lower than that found with ERCP [14]. The routine use of secretin stimulation may further improve the test’s accuracy for identifying pancreatic duct disruption [11]. This result agrees with that of Akisik et al. [26], who found that the sensitivity of secretin-enhanced MRCP for showing extravasation at the site of pancreatic duct disconnection is lower than that of ERCP [23].

The APACHE II score has the advantage of reﬂecting systemic complications. The higher the APACHE II score, the worse the patient’s general condition, especially when the APACHE II score is greater than eight [21]. MRI is a reliable method for determining the severity of acute pancreatitis and has predictive value for the prognosis of the disease [6]. The MRSI has a better ability to predict local complications and disease prognosis. Arvanitakis et al. [6] consider MRI a reliable method for staging the severity of acute pancreatitis. In this study, the number of MPD segments visualized by MRI was negatively correlated with the MRSI score and the APACHE II score. We found that the prevalence of pancreatic duct disruption was correlated with the severity of acute pancreatitis, as determined by the MRSI and APACHE II scores. Our results indicate that the prevalences of MPD integrity and pancreatic duct disruption determined by MRI are associated with acute pancreatitis and thus may become supplementary factors for determining the severity of acute pancreatitis.

Our study has a number of limitations. The first limitation is that pancreatic duct disruption is not reflected by ERCP. There may be false-negative or false-positive results. To avoid these false results, two radiologists experienced in interpreting abdominal images carefully reviewed the images and compared them with a reference image. The second limitation is that the time interval between the MRI and the onset of acute pancreatitis was variable, which may have affected the state of the MPD and the MRSI score. We performed MRI within 48 h after admission to minimize this variability. A third limitation is that coronal and axial SSFSE T2-weighted images are theoretically not good options for patients who breathe rapidly and irregularly. In the present study, most of the patients generally breathed regularly, and we observed the pancreatic duct on the FRFSE T2-weighted images, the coronal and axial SSFSE T2-weighted images, and MRCP images. A forth limitation is that the 12 common physiological and laboratory values that are used to determine the APACHE II score were measured by several physicians and nurses, which may have led to variations between the different observers. However, this variation likely did not affect the observations of the changes in the MPD on MRI or the primary results of the study.

In summary, the pancreatic duct in patients with acute pancreatitis is of normal diameter. The number of main pancreatic duct segments visible by MRI in acute pancreatitis patients and the prevalence of pancreatic duct disruption on MR images in severe acute pancreatitis patients may be supplementary indicators for determining the severity of acute pancreatitis.
